# The Effect of the Timing of Umbilical Cord Clamping on Hemoglobin Levels, Neonatal Outcomes and Developmental Status in Infants at 4 Months Old

**Published:** 2019

**Authors:** Soheila NOURAIE, Sedigheh AMIRALIl AKBARI, Roshanak VAMEGHI, Alireza AKBARZADE BAGHBAN

**Affiliations:** 1 Department of Midwifery, School of Nursing and Midwifery, Shahid Beheshti University of Medical Sciences, Tehran, Iran; 2 Pediatric Neurorehabilitation Research Center, University of Social Welfare and Rehabilitation Sciences, Tehran, Iran; 3 Proteomics Research Center, Department of Basic Sciences, School of Rehabilitation, Shahid Beheshti University of Medical Sciences, Tehran, Iran

**Keywords:** Umbilical cord clamping, Hemoglobin, Child development

## Abstract

**Objectives:**

Delayed umbilical cord clamping (DCC) increases blood transfer to newborns. Hence we investigated the effect of the timing of DCC on hemoglobin levels, neonatal outcomes and developmental status in infants at four months old.

**Materials & Methods:**

This clinical trial examined infants born to 400 pregnant women immediately upon birth and at the age of four months in Isfahan, central Iran in 2016. A table of random numbers was used to randomly allocate the newborns to intervention group with a 90-120-sec delay in umbilical cord clamping and the control group with a clamping delay of below 60 sec, and blood samples were taken from their umbilical cords. The Ages and Stages Questionnaire was used to evaluate the infants’ developmental status.

**Results:**

Umbilical cord hemoglobin was significantly higher in the intervention group compared to in the controls (*P*=0.024). No significant differences were observed between the two groups in terms of neonatal complications except neonatal jaundice was significantly more common in the intervention group (*P*=0.025), although the need for phototherapy was not different between the groups. Overall, no significant differences were observed between the two groups in terms of developmental status at four months old; however, the infants had better problem-solving skills in the DCC group (*P*=0.015).

**Conclusion:**

Despite elevating hemoglobin, DCC has no effects on infant development except in terms of problem-solving skills. Further studies are recommended on the effects of DCC on infant development.

## Introduction

Despite the medical advances in the diagnosis and treatment of diseases, developmental delays in children are still considered a global health challenge in both developed and developing countries (1). Developmental delay generally refers to children who fail to achieve their age-appropriate developmental milestones. Developmental and behavioral problems are the most common pediatric disorders, following infection and trauma (2). Around 200 million children across the world are estimated to not have accomplished or to not be on the path of accomplishing their age-appropriate development (3). This condition is highly prevalent even in developed countries, but does not have a uniform prevalence throughout the world; in high-risk populations, its prevalence can reach as high as 30% (4). The prevalence of developmental and behavioral disorders in children was reported as 15%-20% in the US, 8% in Bangladesh, 15% in Pakistan, 1.5-2.5% in India in infants under age two, up to 10% in Iraq, 3.3% in Brazil and 12.5% in the Netherlands (5-8). In Iran, the prevalence was estimated as 18.7% in Isfahan, 22.5% in Qazvin, 19.8% in Karaj, and 18% in Tehran (2, 9, 10,11). 

Developmental delays in children impose heavy diagnosis, treatment, and health care costs and comprise a major challenge for families and healthcare workers in terms of the time, energy and equipment required. The special educational needs of these children and the costs of specialized schools also constitute another problem for health and education systems (5, 7).

The main reasons for developmental disorders still remain unknown and most cases are multifactorial (12). Infant’s development is affected by different factors, including pregnancy, hereditary, biological, psychosocial and environmental factors. In other words, human development depends on a dynamic and continuous interaction between biological and acquired factors (5). Iron deficiency anemia is associated with developmental disorders in children (13). Iron deficiency affects language, cognitive, socio-emotional and sensory development in children and associated with various neurocognitive impairments (14, 15). Children with chronic anemia during infancy received lower motor scores compared to children with proper iron levels (16). High hemoglobin level upon birth constitutes an iron reserve for dealing with iron deficiency anemia in infants, which accounts for 30%-50% of pregnancy complications in developing countries (10, 17, 18). In Iran, 20.2% of pregnant women are affected by iron deficiency anemia (19, 20). 

Hemoglobin and hematocrit levels are affected by different factors in infants. Delayed umbilical cord clamping (DCC) increases hemoglobin levels in newborns (21-23). If the placenta is cut or separated, and if a fetal vessel is ruptured or punctured or if the newborn is held significantly above the level of the placenta for a while before the umbilical cord clamping, his hemoglobin level may drop after birth. Newborns with DCC have a 32% higher blood volume compared to those with early umbilical cord clamping (22). Serum ferritin is also higher in children with DCC at 6 months old (24). Despite some evidence about the timing of cord clamping, early umbilical cord clamping is still preferred even in the US. DCC helps stabilize blood circulation and reduce the need for blood transfusion and intraventricular hemorrhage in premature infants.

 DCC can be associated with a reduced risk of iron deficiency anemia in term infants and affect neonatal iron storage and neurodevelopment in the long term (25, 26). Hemoglobin levels are significantly lower in newborns with their umbilical cord clamped within less than one min from birth 24-48 h after birth compared to those with DCC. The risk of iron deficiency was two times higher in these children at age three to six months (27). 

Given the importance and prevalence of developmental disorders, the diversity of scientific findings on their contributing factors, the lack of accurate information about the risk factors associated with these problems, especially findings based on clinical trials, the continuing controversy over the timing of umbilical cord clamping, the existing evidence on the effectiveness of DCC in increasing infants' level of hemoglobin, the evidence on iron contributing to children’s development and the lack of studies addressing the subject and since no studies have so far been conducted in Iran on the relationship between the timing of umbilical cord clamping and children’s development with the assumption that hemoglobin levels affect infant development, the present study was conducted to determine the effect of the timing of umbilical cord clamping on infants' hemoglobin levels, neonatal outcomes and development status at four months old. 

## Materials & Methods

The Ethics Committee of Shahid Beheshti University of Medical Sciences, Tehran, Iran approved this study with the code of SBMU.Rec.1393.631. The study was registered in the Iranian Registry of Clinical Trial with the code of IRCT201702066807N19.

The study was conducted upon birth and at four months old on infants born to 400 pregnant women presenting to a healthcare center affiliated to Isfahan University of Medical Sciences and Health Services, Isfahan, Iran in 2016. The inclusion criteria consisted of being of Iranian nationality, age 18 to 35 months, and the mother's parity being four or less. 

The eligible mothers had no pregnancy complications such as diabetes, cardiovascular and renal-pulmonary diseases, preeclampsia, placental abruption and polyhydramnios. The mothers’ most recent delivery had not required the use of forceps or vacuum extractors and was not accompanied with complications such as hemorrhage, dystocia or prolonged labor. In addition, there was no history of known developmental (genetic) disorders or congenital anomalies in either parent’s families. The infants were also not born through delayed or preterm birth, their birth Apgar score was at least 7, their birth weight was above 2.5 kg and their physical health was confirmed at four months old by a physician of the health center. After gaining accommodation letter, the researcher visited the research setting and briefed the mothers on the study objectives. 

After ensuring of the mothers' eligibility and obtaining their informed consent for participation, the researcher proceeded to sampling. The infants were randomly assigned to two groups. The timing of umbilical cord clamping was 90-120 sec after birth in the intervention group and less than 60 sec in the control group. Blood samples were then taken from the infants’ umbilical cord vein and transferred to the laboratory for measuring their hemoglobin level. All the tests were performed by a laboratory technician using the same device. The Ages and Stages Questionnaire (ASQ) was used to examine the infants’ developmental status at four months old. 

The data collection tools consisted of information sheets including demographic, medical, pregnancy and childbirth data and forms associated with the eligibility criteria and the infants' birth profile. A spectrophotometer was used for measuring the infants’ hemoglobin level. To assess the children's developmental status, the literate parents were asked to complete the ASQ. The validity of the information sheets was confirmed using the content validity method and their reliability was assessed using test-retest. The spectrophotometer was calibrated and its validity was confirmed by the manufacturer. Its reliability was also confirmed using test-retest. The ASQ is a globally valid tool with a validity of 83% and a reliability of 90%. It was also adapted and normalized in Iran in 2002-2007 and its concurrent validity was reported as 84%, its reliability as 94% using test-retest and its competence for detecting developmental disorders as over 96% (28). Nonetheless, after diagnosing the infants with developmental delays, the researcher referred them to the center’s physician to ensure of their diagnosis. The data collected were analyzed using SPSS ver.19 (Chicago, IL, USA).

## Results

The results obtained showed no significant differences between the two groups in terms of the mothers’ mean age (Table 1).

**Table 1:** Comparison of the demographic details in the intervention and control groups

Groups		Intervention Group N=200	Control Group N=200	*P*-value
Variables				
Mean &SD of Mother age		28.79±5.07	29.65±5.32	0.521
Mean &SD of Father age		33.98±5.26	34.91±5.88	0.487
Primary	Level of Mother education Frequency(percent)	6(3)	7(3.5)	0.142
High school		135(67.5)	139(69.5)	
Diploma		37(18.5)	36(18)	
College		22(11)	18(9)	
Primary	Level of Father education Frequency(percent)	164(82)	7(3.5)	0.176
High school		36(18)	122(61)	
Diploma		98(49)	43(21.5)	
College		102(51)	28(14)	
Unemployed (Housewife)	Mother’s Employment (frequency/percent)	164(82)	159(79.5)	0.112
Employed		36(18)	41(20.5)	
Boy	Infants' Gender (frequency/percent(	98(49)	101(50.5)	0. 275
Girl		102(51)	99(49.5)	
Mean &SD of birth weight (gr)		3203.51±301.51	3259.32±361.93	0.09
Gestational age(week)		38.78±0.967	38.64±0.81	0.129

The mean and standard deviation of birth Apgar score in the intervention group was 9.82±0/26 and in the controls was 9.74±0/48. No significant differences were observed between two groups.

The mean level of umbilical cord blood hemoglobin was significantly higher in the intervention group compared to in the controls; the infants in the intervention group had developed jaundice more frequently than the controls, although there were no significant differences between the two groups in terms of the need for phototherapy (Table 2). 

**Table 2:** Comparison of umbilical cord blood hemoglobin and neonatal complications in the intervention and control groups

Groups	Intervention Group N=200		Control Group N=200		*P* value
Variables					
umbilical cord blood hemoglobin(g/dl) Mean±SD	15.98±1.44		14.39±1.68		*P*=0.024
160(80)	160(80)		60(30)		*P*=0.023
	Need to Phototherapy		Need to Phototherapy		
	_	+	_	+	0.364
	106(66.3)	54(33.7)	39(65)	21(35)	
Gastrointestinal Infection (frequency/percent)	22(11)		17(8.5)		0.214
Respiratory Infection (frequency/percent)	35(17.5)		39(19.5)		0.116

Studying the infants in terms of the different dimensions of their development at four months old showed problem-solving to be the only dimension in which the two groups differed, as the intervention group received higher scores for it (*P*=0.015) nevertheless, no significant differences were observed in terms of the other dimensions and the overall score (Table 3). 

**Table 3:** Comparison of the infants’ scores for the different dimensions of development in the intervention and control groups

Groups	Intervention Group N=200	Control Group N=200
Dimensions of development	Mean±SD	Mean±SD
Communication	53.44±9.62	51.24±8.58
Gross motor	52.41±6.44	53.32±5.33
Fine motor	49.25±7.25	48.21±6.98
Problem-solving	54.41±5.43	41.98±6.32
Personal-social	48.32±8.22	49.34±7.94

## Discussion

We should that DCC could positively affect the hemoglobin level in infants. The relationship between infants' hemoglobin level 24-48 h was investigated after birth and the timing of umbilical cord clamping in them (27). Moreover, higher hemoglobin levels were revealed in the DCC group (29), which was consistent with the present findings. Higher hematocrit was reported at 4 h of birth in DCC group (30).

No significant differences were found between the two groups examined in terms of the effect of the timing of umbilical cord clamping on hemoglobin levels, although serum ferritin levels were 45% higher in the DCC group than in the early clamping group, suggesting a statistically significant difference; however, no cases of iron deficiency anemia in these groups were observed (31). Early umbilical cord clamping could reduce hemoglobin levels in infants at two months old and that a delay in clamping, even by two minutes, can increase iron reserves in term infants and thus improve their blood status before age six months (32). The timing of umbilical cord clamping had a significant effect on blood volume and red blood cell mass and that holding the infant below the level of the placenta and a few minutes of delay in clamping the umbilical cord can increase the infant’s blood volume by 40% (33). Iron plays a role in many central nervous system functions in fetuses and infants. The main effect of iron is on the myelination process and the main ganglia functions and it also reduces neurometabolic activity (15, 16). Iron deficiency negatively affects the function of iron-dependent enzymes in all cells and contributes significantly to the function of neurotransmitters and muscles (10, 16). DCC was effective in improving hemoglobin levels and anemia status (34). DCC has been shown to improve iron stores in infants to 6 months of age (35).

The present study found no significant differences between the two groups in their dimensions of development, including communication, gross and fine motor skills and personal-social development; however, they differed in terms of problem-solving. A clinical trial was conducted to examine the effect of delayed umbilical cord clamping on infants’ development at four months old and found better problem-solving skills in the delayed umbilical cord clamping group, which is consistent with the present findings (36). The Ages and Stages Questionnaire scores had no differences between late and early clamping (27).

Anemia affects the communication aspect of development and causes delays in it (9, 37), while Perez et al. found this relationship insignificant (38). A significant relationship was found between anemia and motor delays (16, 38). Hemoglobin levels did not affect the personal-social dimension of development (9), which is consistent with the present findings.

Infants in the intervention group had developed jaundice more frequently than the controls, although there were no significant differences between the two groups in terms of the need for phototherapy. However, the amount of required phototherapy for jaundice in the early cord clamping group was less than the late cord clamping group (27). No difference was reported in hyperbilirubinemia between two groups (29).

The present study found higher mean Apgar scores in the intervention group compared to in the controls, although the difference was not statistically significant. There were no significant differences between early and late clamping for the neonate’s Apgar score less than seven at five minutes (27, 29). Significant differences were observed between the early clamping group (30 sec after birth) and the delayed clamping group (3 min after birth) in terms of the 1-min and 5-min Apgar scores (33). Besides, significantly higher 5-min Apgar scores were found in the group of preterm infants with DCC compared to in the early clamping group (39), but no significant the Apgar score was reported at 5 min between two groups (40).

**In conclusion, **delays in umbilical cord clamping can significantly elevate umbilical cord hemoglobin levels; however, it also increases the risk of neonatal jaundice and has no effects on developmental status, except in the problem-solving dimension. Further studies are recommended on this subject. 
